# Intravital electrochemical nanosensor as a tool for the measurement of reactive oxygen/nitrogen species in liver diseases

**DOI:** 10.1186/s12951-022-01688-z

**Published:** 2022-11-24

**Authors:** Tatiana Abakumova, Alexander Vaneev, Victor Naumenko, Arina Shokhina, Vsevolod Belousov, Arsen Mikaelyan, Kamilla Balysheva, Peter Gorelkin, Alexander Erofeev, Timofei Zatsepin

**Affiliations:** 1 Skolkovo Institute of Science and Technology, Bolshoy Boulevard, 30/1, Moscow, 121205 Russia; 2 National University of Science and Technology «MISIS», Leninskiy Avenue, 4, Moscow, 119049 Russia; 3 V. Serbsky National Medical Research Center for Psychiatry and Narcology, Kropotkinskii Lane, 23, Moscow, 117034 Russia; 4 Federal Center of Brain Research and Neurotechnologies of the Federal Medical Biological Agency, Ostrovityanova Street, 1/10, Moscow, 117513 Russia; 5 Koltzov Institute of Developmental Biology of Russian Academy of Sciences, Vavilova Street, 26, Moscow, 119334 Russia; 6 Pirogov Russian National Research Medical University, Ostrovityanova, 1, Moscow, 117997 Russia; 7 Lomonosov Moscow State University, Leninskie Gory, Moscow, 119991 Russia

**Keywords:** Oxidative stress, Intravital imaging, Electrochemical detection, Nanosensors, Liver disease, Hepatocellular carcinoma

## Abstract

Reactive oxygen/nitrogen species (ROS/RNS) are formed during normal cellular metabolism and contribute to its regulation, while many pathological processes are associated with ROS/RNS imbalances. Modern methods for measuring ROS/RNS are mainly based on the use of inducible fluorescent dyes and protein-based sensors, which have several disadvantages for in vivo use. Intravital electrochemical nanosensors can be used to quantify ROS/RNS with high sensitivity without exogenous tracers and allow dynamic ROS/RNS measurements in vivo. Here, we developed a method for quantifying total ROS/RNS levels in the liver and evaluated our setup in live mice using three common models of liver disease associated with ROS activation: acute liver injury with CCl_4_, partial hepatectomy (HE), and induced hepatocellular carcinoma (HCC). We have demonstrated using intravital electrochemical detection that any exposure to the peritoneum in vivo leads to an increase in total ROS/RNS levels, from a slight increase to an explosion, depending on the procedure. Analysis of the total ROS/RNS level in a partial hepatectomy model revealed oxidative stress, both in mice 24 h after HE and in sham-operated mice. We quantified dose-dependent ROS/RNS production in CCl_4_-induced injury with underlying neutrophil infiltration and cell death. We expect that in vivo electrochemical measurements of reactive oxygen/nitrogen species in the liver may become a routine approach that provides valuable data in research and preclinical studies.

**Supplementary Information:**

The online version contains supplementary material available at 10.1186/s12951-022-01688-z.

## Introduction

Reactive oxygen/nitrogen species (ROS/RNS) are highly reactive molecules involved in cell and tissue metabolism and play signaling roles in physiological (cell proliferation, apoptosis) and pathophysiological processes (inflammation, cancer) [1]. Moderate levels of free radicals can modulate the activity of various transcription factors and protein kinases and regulate cell differentiation, autophagy, and apoptosis [2, 3]. An imbalance in ROS/RNS production and antioxidant defense systems leads to an excessive level of ROS/RNS, called oxidative stress, which causes cell damage and contributes to the development of various pathological conditions [4]. ROS/RNS are also involved in the mechanisms of the immune response. For example, ROS growth can be observed in activated phagocytes after exposure to pathogens [1]. Cancer cells have increased ROS/RNS levels due to increased metabolism, so tumor progression is closely related to increased ROS/RNS levels. Moreover, conventional drugs, such as the chemotherapy drugs cisplatin and doxorubicin, can cause increased production of ROS/RNS, and conversely, ROS/RNS can interfere with drug efficacy due to increased degradation of the molecule.

Measurement of ROS/RNS levels is an effective tool in drug development, diagnosis and monitoring of various diseases. In addition, ROS/RNS imaging can be useful in regenerative medicine to analyze cell proliferation and differentiation. To measure ROS/RNS in tissues, several methods have been developed, most of which are implemented for ex vivo use: EPR spectroscopy [5], various fluorescent dyes induced by ROS (dichlorofluorescein, hydrocyanine [6], etc.), luminol and its derivatives, cytochrome C, free glutathione fraction analysis and other approaches for indirect ROS/RNS measurements. Supplementary Table S1 summarizes the data on the current methods that are used to assess markers of oxidative stress. EPR spectroscopy is a sensitive method for ROS and RNS detection, but it is also laborious and cannot be implemented for in vivo use. Direct or indirect observations of the formation of superoxide radicals are usually carried out using spin traps [5]. The majority of current methods are focused on fluorescent and chemiluminescent dyes and have become valuable tools for ROS measurement in cell culture and for histological staining [7]. Unfortunately, these methods have a number of limitations, including interference with compounds that could affect fluorescence intensity [8]. Additionally, they are not applicable for dynamic measurements of ROS/RNS changes in vivo, since a decrease in ROS/RNS will not lead to changes in fluorescence intensity. Another ROS/RNS sensor that has recently been introduced in vivo is a genetically encoded protein that is sensitive to the presence of hydrogen peroxide (HyPer). Various compartment-specific variants of Hyper (nucleus, cytoplasm, etc.) have been studied for the real-time imaging of cells and small organisms (e.g., zebrafish larvae) [9]. However, in vivo detection of ROS/RNS in live animals is still challenging for most animal models due to poor targeted delivery of dyes/DNA/proteins in vivo and limited tissue transparency in the UV–visible region, as conventional fluorescence microscopy/tomography limits the available depth to 100 µm. Moreover, the HyPer fluorescence spectrum (excitation 420/500 nm, emission 516 nm) overlaps with the autofluorescence region of tissues, which reduces sensitivity in whole-body experiments.

Primary ROS/RNS, hydrogen peroxide, nitric oxide, peroxynitrite and nitrite ions can be directly oxidized or reduced using fixed electrochemical potentials. Consequently, ROS/RNS can be detected and quantified using electroanalytical methods, which also provide the ability to map analytes with high resolution and high sensitivity. Electrochemical methods have proven to be very useful for the quantitative determination of analytes in vivo due to their several advantages [10].

First, the electrochemical measurement of ROS/RNS is direct and occurs in label-free mode. Second, electrochemical methods allow the measurement of ROS/RNS with a short half-life. Third, electrochemistry allows real-time monitoring of ROS/RNS with high temporal resolution. Traditional electrodes have a large surface area and are poorly compatible with accurate measurements in the abdominal cavity, while recently developed small nanoelectrodes based on nanopipettes [11–13] can overcome these limitations. Nanopipettes are used to visualize biological objects using a scanning ion-conductance microscope [14], quantitative nanomechanical mapping of cells [15], dynamic mapping of extracellular pH at the single-cell level [16], and determination of ROS/RNS generation by anticancer drugs [17–20]. Recently, disk-shaped carbon nanoelectrodes (UNEs) have been used to determine ROS/RNS [11, 16, 21–24] and oxygen [7] within a single cell. Due to their extremely small dimensions in the nanometer range, these electrodes can penetrate the cell membrane without compromising the integrity and viability of cells. At the same time, these electrodes are strong enough to penetrate the tumor tissue for subsequent in vivo measurements [17]. Thus, electrochemical sensors are a very promising tool for in vivo quantification of ROS/RNS and can replace traditional approaches [25–27].

Previously, we have shown that we are able to detect total ROS/RNS level in tumor tissue after anticancer treatment [21]. Although ROS detection in subcutaneous tumors is a very helpful tool for preclinical studies [28], it is still challenging to detect ROS in abdominal organs, including orthotopic tumor models. Organs and tissues differ in anatomical structure, iron concentration, vascularization and other parameters, all of which can impact ROS detection.

The liver is a major organ that is regularly attacked by ROS, and real-time measurement of ROS/RNS in the liver will help in research and preclinical studies of liver diseases. Different pathological processes in the liver are associated with ROS/RNS imbalance: cell death (toxic injury), intense proliferation (hepatectomy), and carcinogenesis (hepatocellular carcinoma) and can be analyzed and quantitatively measured. In this study, we focused on the evaluation of electrochemical nanosensors as a tool for quantifying total ROS/RNS level in the liver in vivo using general models of liver diseases. We propose to use an approach that has high sensitivity and low invasiveness. An important advantage of this method is the use of a nanoelectrode that allows label-free measurements in the inner layers of the liver. Liver phenotypes after partial hepatectomy, induced hepatocellular carcinoma, and acute liver injury with CCl_4_ were characterized by real-time quantitative PCR (RT-qPCR), serum ALT/AST levels, intravital microscopy, and histological staining and further studied by electrochemical microscopy. Taken together, this is the first work in which we demonstrated intravital measurement of total ROS/RNS level in damaged livers in living animals using electrochemical nanosensors. Despite the different nature of the underlying processes, we have demonstrated that the production of ROS/RNS in the liver can be directly quantified using electrochemical sensors in a living animal.

## Materials and methods

### Instrumentation

All electrochemical measurements were carried out at room temperature using a two-electrode configuration with an AgCl electrode as the counter-reference electrode (a 0.3 mm AgCl-coated Ag wire); all potentials are reported *vs* Ag/AgCl reference. In vitro*/*in vivo voltammetric experiments were performed at room temperature (24 ± 2 °C) inside a Faraday cage. The Faradaic current was measured with a MultiClamp 700B patch-clamp amplifier (Molecular Devices, USA). Transfer and recording of measurements to a computer were carried out using the ADC-DAC converter Axon Digidata 1440B (Molecular Devices, USA) and pClamp 10 software. The micromanipulator PatchStar (Scientifica, UK) was used to feed the nanosensor. The current signals were filtered with 0.5 kHz lowpass filters. The principal scheme of the experiment is shown in Additional file 1: Figure S1.

### Nanoelectrode fabrication

**Scheme 1:**
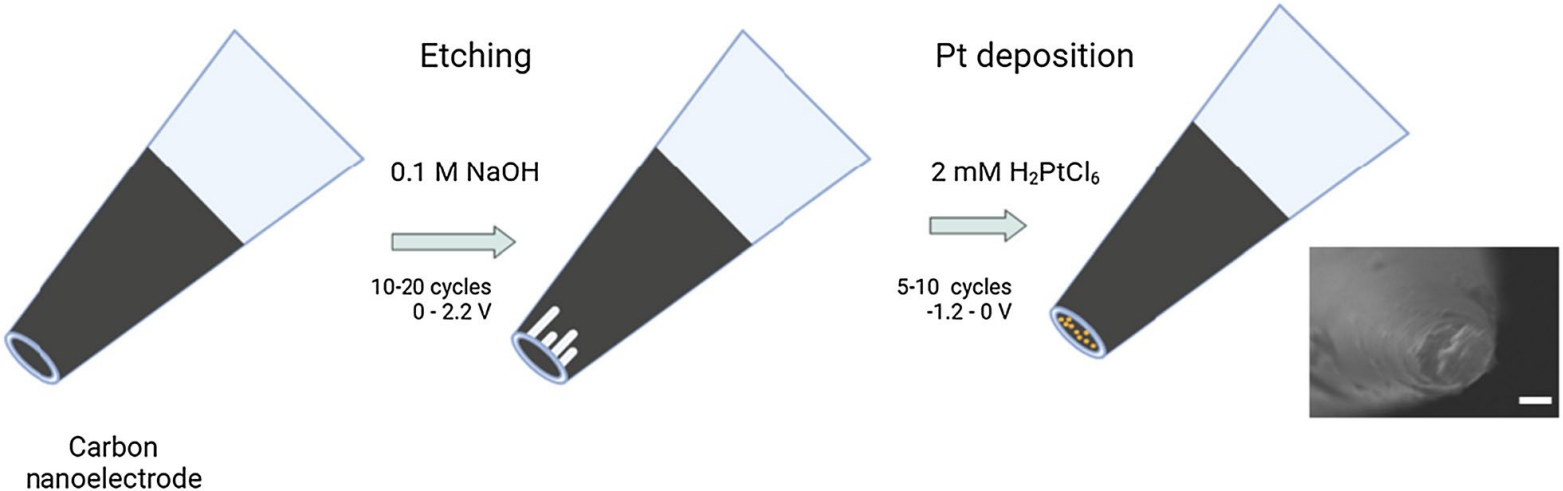
Scheme of the fabrication of the platinized nanoelectrode. The inset shows an SEM microphotograph of the nanoelectrode. Scale bar, 500 nm

Platinized nanoelectrodes (PtNEs) were prepared based on commercially available disk-shaped carbon nanoelectrodes (CNEs) isolated in quartz (ICAPPIC Limited, UK) with diameters of 50–150 nm. The preparation of CNE has been described in detail previously [21, 24]. A scheme of the fabrication of the platinized nanoelectrode is shown in Scheme 1. Briefly, a platinum electrode is a nanopipette filled with carbon. The CNEs were initially placed in 1 mM ferrocene methanol in PBS buffer to verify their operability for further work. The initial electrode radius (r) was estimated using the steady-state current (i_ss_) at 0.4 V vs. Ag/AgCl in 1 mM ferrocene methanol in PBS buffer according to the formula i_ss_ = 4.64*r*F*c*D, where F is the Faraday constant, c is the concentration and D is the diffusion coefficient (7.8 × 10^−6^ cm^2^ s^−1^ for FcCH_2_OH) [29] **(**Additional file 1: Figure S2A). To enhance the adhesion of platinum on the surface, a nanocavity etched into the carbon electrode was used. Electrochemical etching was performed by means of cyclic voltammetry (CV) from 0 to 2.2 V in 0.1 M KOH, 10 mM KCl for typically 15–40 cycles until the formation of nanocavities (Additional file 1: Figure S2B) As a result of etching, two peaks appear on the voltammogram corresponding to the complete oxidation of FcCH_2_OH and reduction of ferrocenium inside the nanocavity**.** Then, platinum was deposited to increase the electrochemical activity of the surface (Additional file 1: Figure S2C). Electrochemical deposition of platinum was achieved by cycling from 0 to − 0.8 V with a scan rate of 200 mV s^−1^ for 4−5 cycles in 2 mM H_2_PtCl_6_ solution in 0.1 M hydrochloric acid. To assess the deposition of platinum on the end of the nanoelectrode and evaluate the diameter of PtNE, we recorded CV in 1 mM FcCH_2_OH. The diameter of the PtNE was approximately 50 − 150 nm, which shows excellent electrochemical performance. We performed a study to investigate the reproducibility of the nanoelectrode fabrication by preparing several nanopipettes (N = 5) with identical pulling parameters, depositing carbon, and averaging their steady state (Additional file 1: Figure S2D). It was shown that PtNEs have high reproducibility. The total concentration of ROS/RNS was evaluated at a potential of + 800 mV vs. Ag/AgCl. Prior to the measurements, the platinum electrode was calibrated using a series of standard hydrogen peroxide or sodium nitrite solutions (from 10^–7^ M to 10^–4^ M) (Additional file 1: Figures S3, S4).

### Animal models of liver diseases

All *in vivo* experiments were performed in accordance with the Ethical Committee of the Institute of Developmental Biology, Moscow, Russia (Approval № 19), and the following experimental protocols were in accordance with relevant institutional and national guidelines. All mice were divided into cages per group (n=4-6 per group). Water was given *ad libitum.* To minimize animal suffering during experiments, mice were anesthetized with either isoflurane (partial hepatectomy model) or zoletil/xylazine anesthesia (other types of experiments).

#### *Hepatectomy*

Hepatectomy was performed using a method that was previously described with minor modifications [30]. Briefly, mice were anesthetized (2% isoflurane), shaved and sanitized with 70% ethanol. An incision was made along the midline of the abdomen, and the median lobe of the liver was exposed, ligated with a 4-0 silk suture and removed with scissors. After that, the peritoneum was closed with a 4-0 suture needle, and the skin was fixed with 7 mm wound clips. Sham-operated animals were used as controls and underwent all surgical procedures (abdomen opening, sanitation, lobe rotation, wound closure) except liver lobe removal.

#### *Hepatocellular carcinoma*

To induce the development of hepatocellular carcinoma in mice, we injected oncogenic plasmids according to a previously published protocol [3]: 10 µg pT3-EF1a-myr-AKT (Addgene #31789), 10 µg pT3-EF1a-YAP-S127A (Addgene #46049), 10 µg pT3-EF1a-c-Myc (Addgene #92046), and 5 µg pCMV-SB100 (Addgene #34879) per mouse. Briefly, we prepared a mix of plasmids in sterile 0.9% NaCl for each mouse and performed hydrodynamic intravenous injection (100 µl per 1 g of mouse) for 3 groups of mice that represent different stages of HCC development: 3 days, 1 week and 2 weeks after injection.

#### ***CCl***_***4***_***-induced liver injury***

To analyze the ROS/RNS response to toxic agent-induced injury, we injected different doses of carbon tetrachloride intraperitoneally (0.1 ml/kg, 0.05 ml/kg, 0.01 ml/kg in 0.5 ml of olive oil). Mice injected with olive oil or PBS were used as controls (number of animals per group 4-6).

All mice were anesthetized 24 h after injection and analyzed using electrochemical microscopy. After analysis, the blood was collected in EDTA-containing tubes, mice were sacrificed by cervical dislocation, and liver samples were taken for subsequent experiments (RT-qPCR) and histological staining.

### Electrochemical measurements in vivo

Prior to ROS/RNS measurements in vivo, mice were anesthetized using Zotelil (50 mg/kg) and xylazine (5 mg/kg). After that, the abdomen was sanitized with 70% alcohol, an incision was made along the midline (2 cm), and the median liver lobe was exposed without additional cuts of ligaments and skin. The liver was moistened with sterile 0.9% NaCl solution, placed on a pedestal (Petri dish) and moved to the microscope table. The reference electrode was placed under the liver to ensure reliable contact with it and then tested with an electrical circuit.

The nanoelectrode was placed at the surface of the liver using a micromanipulator and then penetrated through the outer layers of the tissue. First, a cyclic voltammogram was recorded (from -800 to + 800 mV, scan rate 400 mV/s) at the initial depth, and then the current was recorded in the system at + 800 mV at a depth of 1000 μm to determine the total ROS/RNS levels. To analyze oxidative stress in the liver during the development of the induced hepatocellular carcinoma, we detected total ROS/RNS levels at depths up to 1000 µm with 100-µm steps in the center of the tumor and at the periphery. Then, a cyclic voltammogram was recorded (from − 800 to + 800 mV, scan speed 400 mV/s) to determine the stability of the electrochemical characteristics of the nanoelectrode. The electrode was removed from the tissue, and cyclic voltammograms were recorded under the same conditions in PBS buffer. The processing was carried out by normalizing the current measured at + 800 mV to the current values at + 800 mV when recording a cyclic voltammogram. Since the size of nanoelectrodes can vary from an electrode to electrode, this internal normalization is necessary for all measurements.

Serum ALT/AST measurement, intravital confocal microscopy, histological staining, RT-qPCR methods, and DCFDA assay are described in the Supplementary Information.

### Statistics

Statistical analysis was performed using GraphPad Prism 8 software. One-way ANOVA with Bonferroni correction was used to analyze the data sets.

## Results

### Preparation of the platinized nanoelectrode and ROS/RNS measurements

**Fig. 1:**
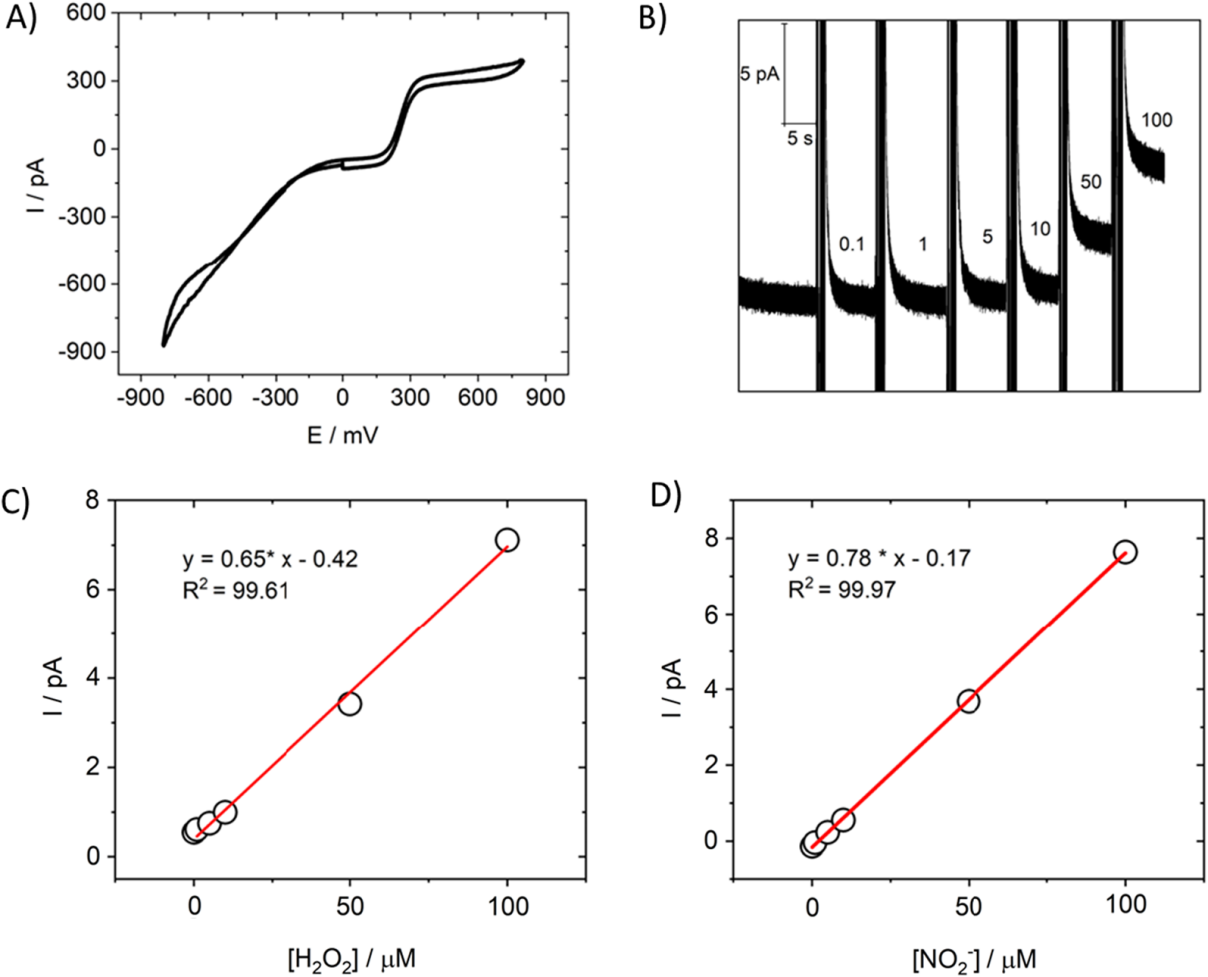
Characteristics of platinized nanoelectrode. **A** Current–voltage characteristic in 1 mM FcMeOH in PBS. Scan rate 400 mV s^−1^. **B** Platinized nanoelectrode calibration. Chronoamperogram of a platinized electrode at + 800 mV, measured in solutions with different concentrations of hydrogen peroxide. The vertical lines in the graph indicate the change in solution concentration. Concentration is shown in micromoles. **C** Calibration curve (current vs H_2_O_2_ concentration at + 800 mV (vs. Ag/AgCl)) showing good linear response **D** Calibration curve (current vs NO_2_^−^ concentration at + 800 mV (vs. Ag/AgCl)) showing good linear response

Detailed information on the fabrication of a nanoelectrode was described in our previous article [21]. For further measurements, we controlled the size of the electrochemically active surface of the Pt nanoelectrode and electrochemical performance by measuring CV in a 1 mM ferrocene methanol solution in PBS (Fig. 1A). We performed measurements in the constant potential mode at + 800 mV vs. Ag/AgCl. First, the platinized electrodes were calibrated using water solutions of hydrogen peroxide and sodium nitrite with physiologically relevant concentrations of 0.1–100 µM (Fig. 1B, 1C, 1D). Hydrogen peroxide and nitrite species are the end-products of many ROS/RNS generated in the cell—the most stable ones in biological environments [31]. The concentration range from ∼1 to 100 μM matches biologically relevant ROS/RNS concentrations expected in the damaged liver tissue [32]. The oxidation potentials of hydrogen peroxide and sodium nitrite fit the entire potential range of ROS/RNS of interest.

### Measurements of total ROS/RNS level in liver tissue in vivo using electrochemical nanosensors

**Fig. 2:**
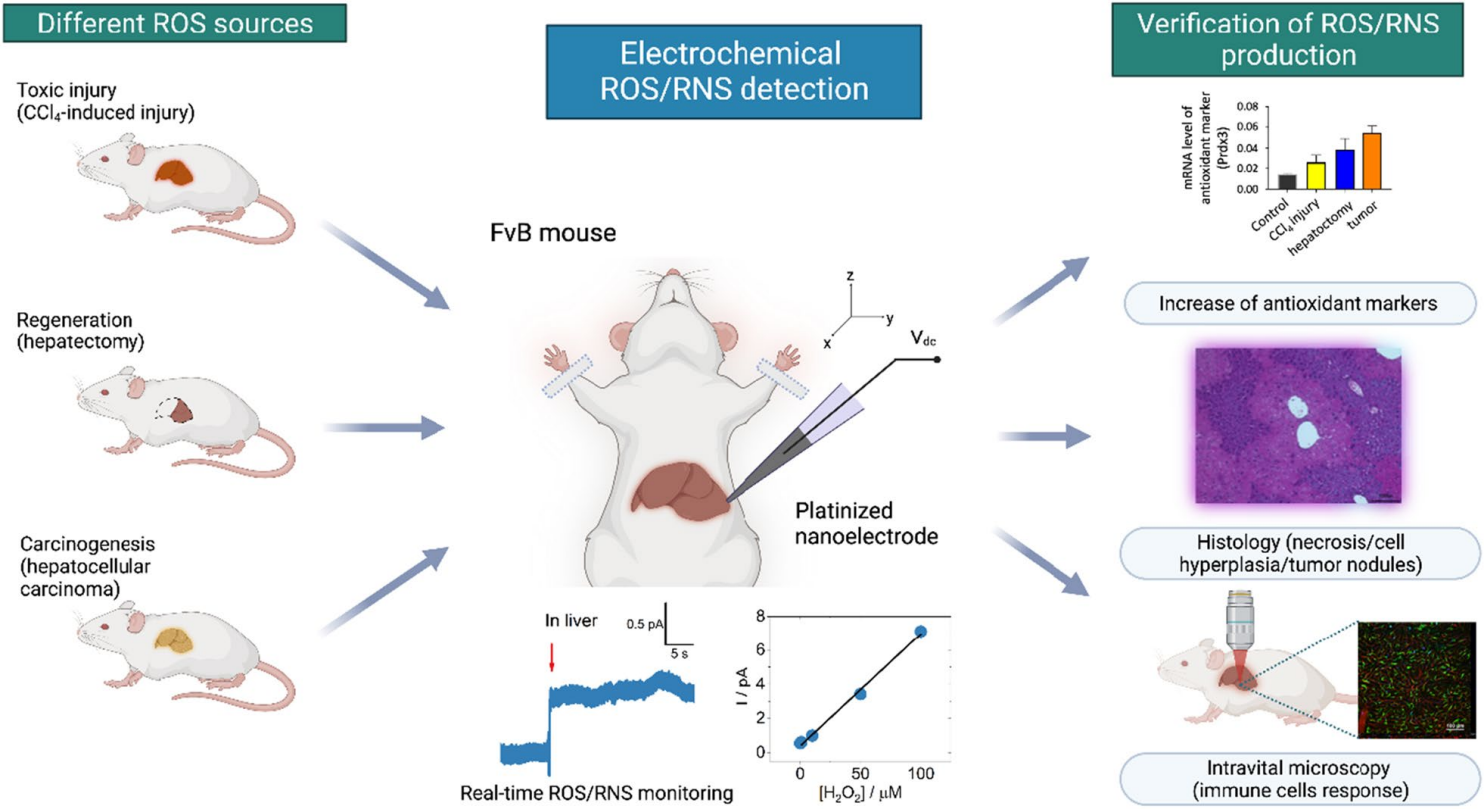
Schematic overview of intravital electrochemical nanoelectrodes for total ROS/RNS level measurement in different pathological processes in the liver: CCl_4_-induced liver injury (toxic injury), hepatectomy (regeneration-associated processes), and development of hepatocellular carcinoma (carcinogenesis). Each pathology was verified and characterized by a complex of methods (expression of antioxidant markers by RT-qPCR, intravital confocal microscopy, histology)

Previously, it was shown that electrochemical nanoelectrodes are able to quantify total ROS/RNS levels in single cells and tumor tissues [21, 22]. The electrocatalytic sensitivity of the Pt-black tip and its selectivity for ROS/RNS allow us to measure ROS/RNS generation in vitro. [33] Measurement of ROS/RNS in each organ and tissue has its own peculiarities: different backgrounds of ROS levels, iron concentrations, blood flow, and mitochondrial activity. Here, we described a fast technique and highly reproducible method for ROS/RNS detection in normal and injured livers, and we verified and characterized it by other methods (Fig. 2). Platinized nanoelectrodes have characteristic steady-state voltammograms for each of the four species composing oxidative bursts to demonstrate sensitivity to all components of oxidative stress and could be applied for intravital ROS/RNS detection.

**Fig. 3:**
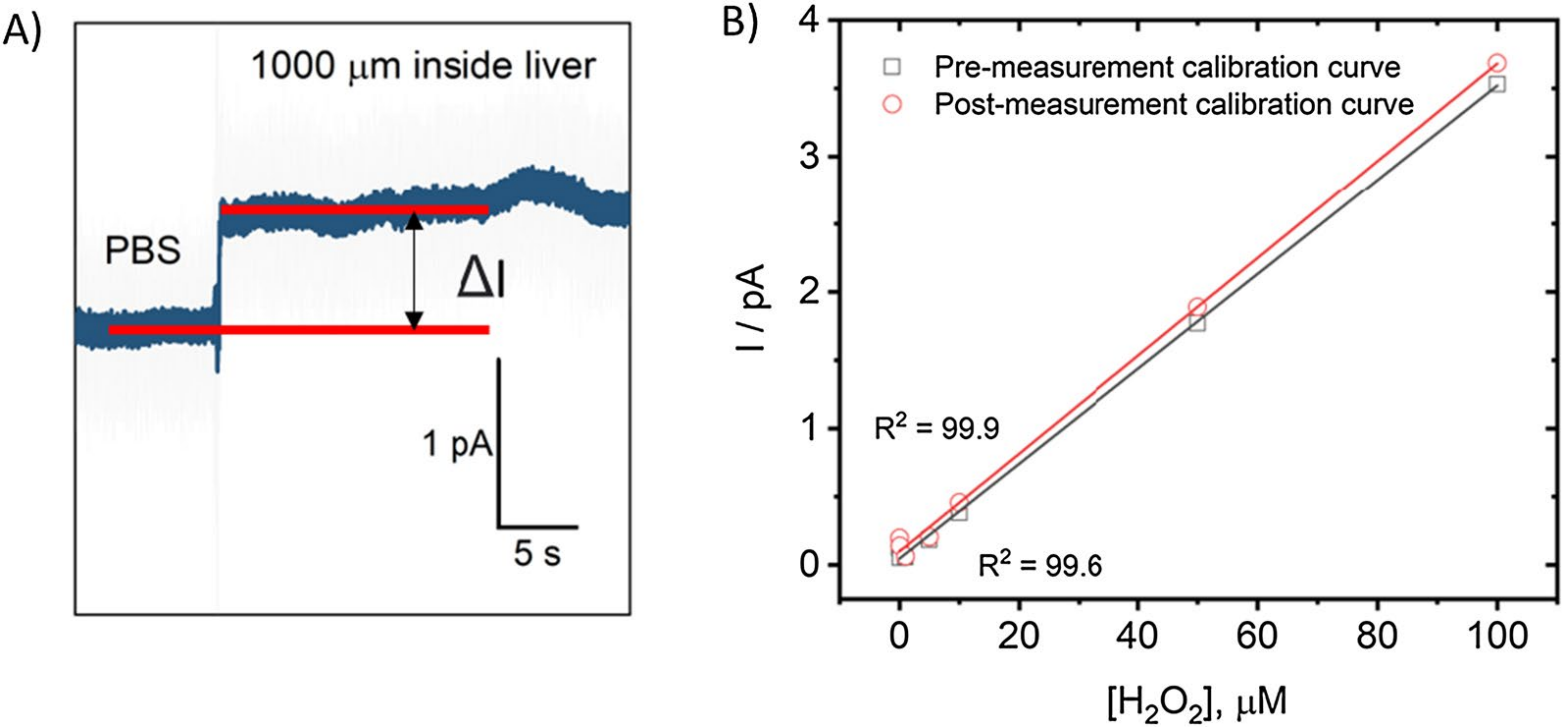
In vivo electrochemical measurements. **A** Amperometric curve recorded inside the liver at 1000 µm. **B** Pre- and post-measurement calibration curve (current vs H_2_O_2_ concentration at + 800 mV (vs. Ag/AgCl)) showing good linear response

We performed measurements inside the liver in amperometry mode. In this case, the current values were measured in solution, on the surface of the liver, and at a depth of 1000 μm. The difference between the current values at a given depth and the values in the solution was taken as the measured signal (Fig. 3A). It should be noted that during the measurements, various electrochemically active species contributed to the electrochemical measurements; therefore, the obtained values were normalized to the values in the control group of mice.

When ROS/RNS are measured in the liver, the electrochemically active area of the electrode can be fouled, which will greatly affect the results of the experiment. Thus, we investigated the stability of nanoelectrodes before and after immersion in a normal homogeneous liver in vivo by calibrating the electrode before and after the experiment (Fig. 3B). The nanoelectrode turned out to be stable during measurements since the calibration curves before and after the experiment were approximately the same. If the electrode was contaminated, we did not take these results into account. We then analyzed the time dependence of the electrochemical signal to address stability issues. We have demonstrated that the signal is stable for at least 40 min, which exceeds the usual measurement time range (5–10 min). Previously, using a nanoelectrode, experiments were performed inside the tumor [21].

Before the experiment, a small incision was made along the midline of the mouse abdomen, exposing the median lobe of the liver (Additional file 1: Fig. S5A).

We also analyzed total ROS/RNS levels in the ex vivo liver (in the lobe of the liver after removal during partial hepatectomy) and found an increased signal compared to in vivo measurements (Additional file 1: Fig. S6). All of these results highlight the importance of the setup for correct in vivo ROS/RNS measurements and confirm the sensitivity of electrochemical detection.

### Oxidative stress in CCl_4_-induced injury

**Fig. 4:**
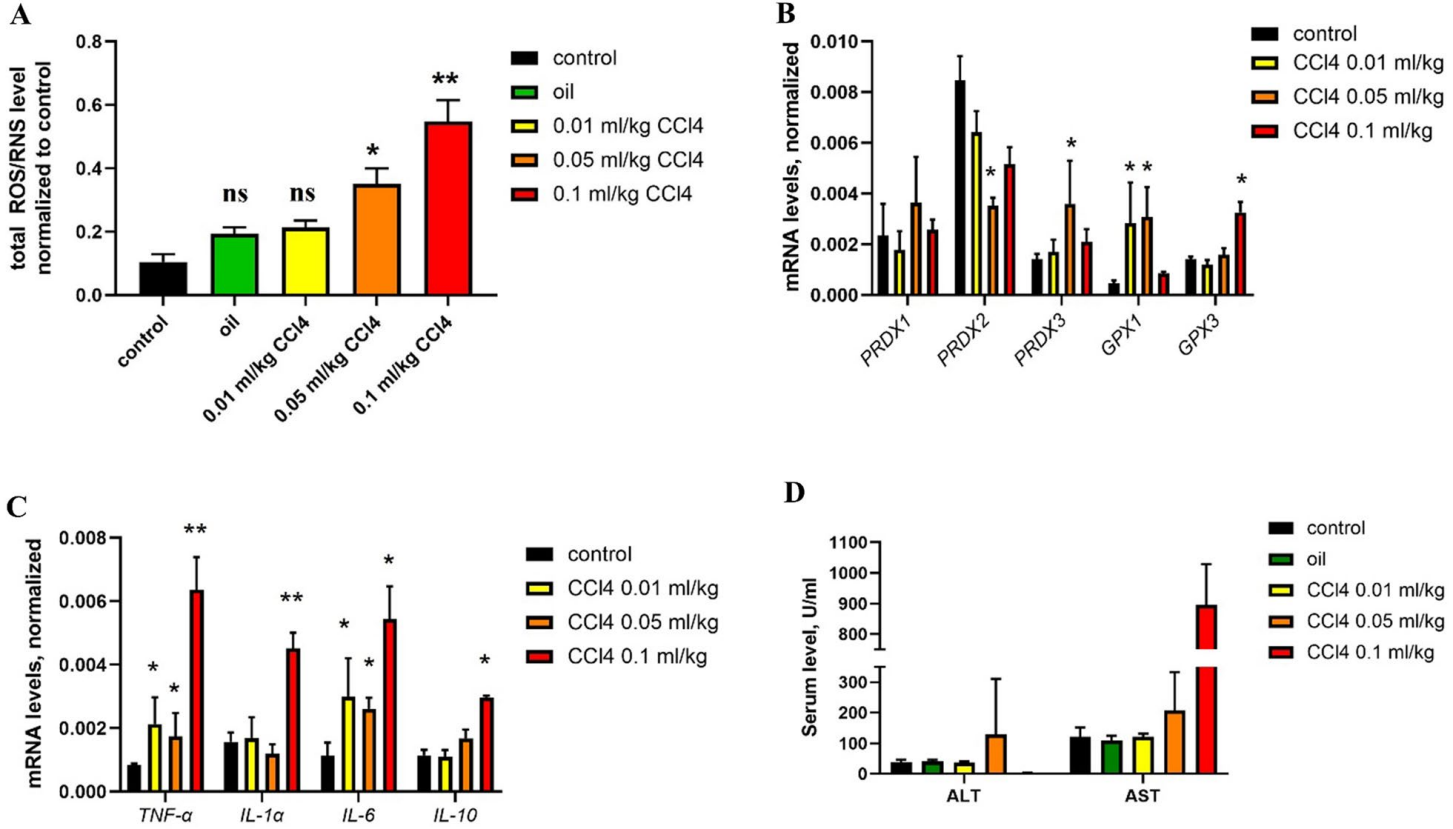
Analysis of oxidative stress in mice after CCl_4_-induced injury. Measurement of total ROS/RNS levels using electrochemical microscopy. **A** Analysis of the expression of antioxidant defense enzymes and **B** proinflammatory cytokines **C** using RT-qPCR and serum ALT/AST levels. **D** Statistical analysis: **A** ** p-value < 0.001, * p-value < 0.005, ns-non-significant. **B**–**D** results show the mean ± SEM

**Fig. 5:**
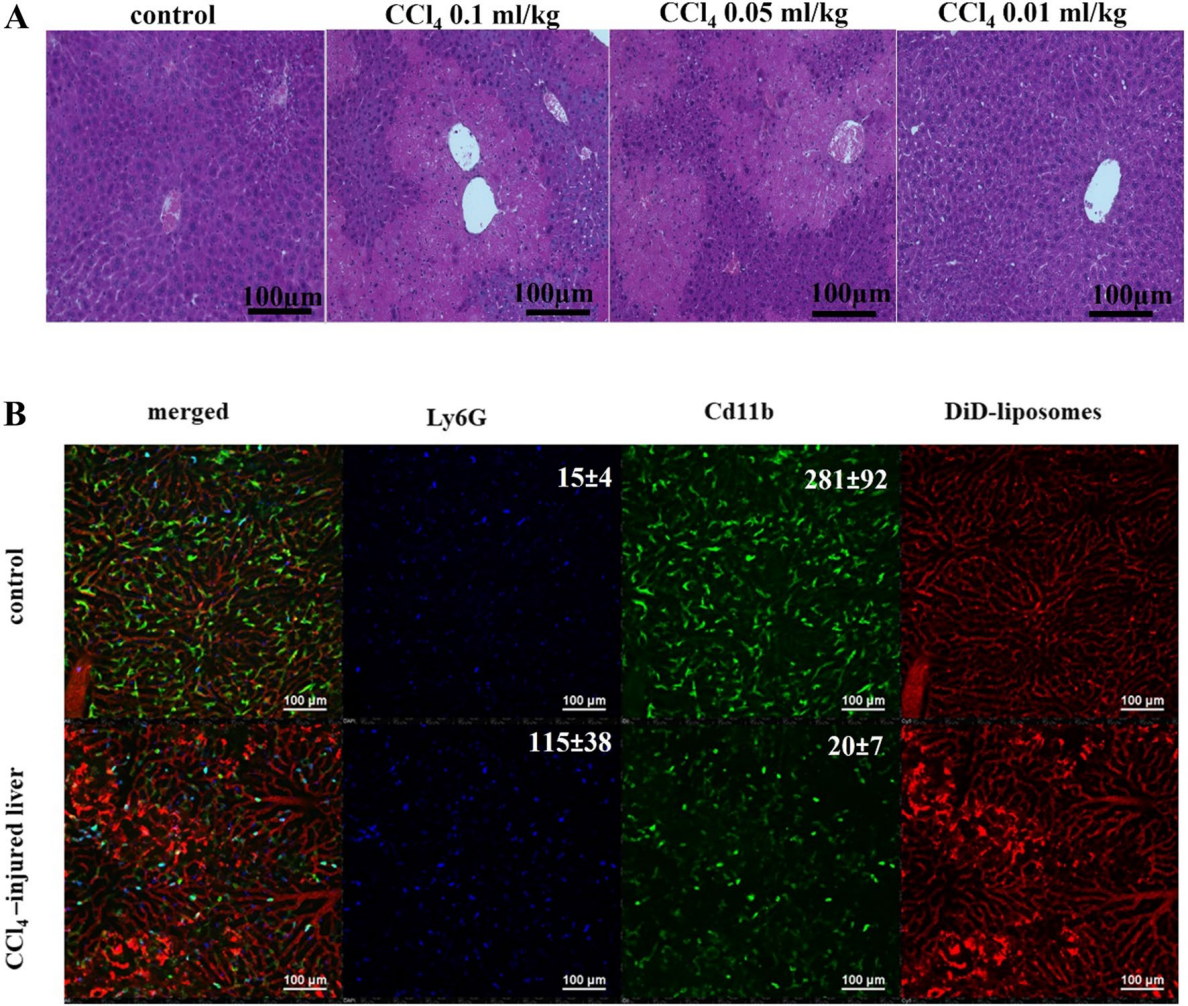
Pathological processes in the CCl_4_-injury model. **A** Histological staining of liver tissue after a single injection of CCl_4_ at different doses (0.1 mg/kg, 0.05 mg/kg and 0.01 mg/kg). Mice injected with oil were used as controls. **B** Intravital imaging of CCl_4_-injured liver 24 hours after injection. Anti-Ly6G antibodies (neutrophils, blue), DiD liposomes (sinusoids, red), anti-CD11b antibodies (Kupffer cells, green). The numbers of Ly6G+ cells and CD11b+ cells per field of view are shown as the mean ± SD

Inhalation or injection of tetrachloride (CCl_4_) is a common approach to induce liver injury that results in elevated ROS/RNS levels in a dose-dependent manner due to massive cell death followed by fibrosis [34]. To analyze the sensitivity of an electrode for ROS/RNS detection in the injured liver, we stimulated dose-dependent ROS/RNS production by i.p. injection of CCl_4_. We used three doses of CCl_4_, 0.01, 0.05 and 0.1 ml/kg, in oil with two types of control, olive oil and phosphate-buffered saline (PBS), and analyzed total ROS/RNS levels in the liver tissue using electrochemical microscopy. Oil and a minimal dose of CCl_4_ (0.01 ml/kg) led to an insignificant increase in total ROS/RNS levels. Further dose escalation (0.05 and 0.1 ml/kg) correlated with elevation of ROS production in the liver (Fig. 4A). To confirm ROS induction, we analyzed changes in the gene expression of the antioxidant defense system (peroxiredoxins, glutathione peroxidases) and markers of inflammation (*TNF-α, IL-1α, IL-6, IL-10*) and measured ALT/AST levels in the serum (Fig. 4B–D). We found that the expression of inflammation markers and serum ALT/AST levels (Fig. 4C, D) had a significant positive correlation with the results of electrochemical microscopy, while analysis of the expression of antioxidant defense components provided rather controversial results (Fig. 4B). If the dose of 0.05 ml/kg was associated with a more than two-fold increase in the expression of antioxidant defense and inflammation markers, the 0.1 ml/kg dose did not change the expression of *PRDXs 1–3* and *GPX1-3*. This result can be driven by the suppression of cell metabolism in the liver at high doses of CCl_4_. Serum analysis revealed a correlation between increased ALT/AST levels and higher doses of CCl_4_ (Fig. 4D). To confirm the action of CCl_4_, we performed histological analysis of these liver samples (hematoxylin and eosin staining) and revealed large areas of necrosis after injection of CCl_4_ at high doses (0.05 ml/kg and 0.1 ml/kg) (Fig. 5). At a dose of 0.1 ml/kg, we also observed a drop in the number of CD11b + cells and significant neutrophil infiltration (Fig. 5A), which can be a result of the massive necrosis of Kupffer cells and their elimination by the immune system. Thus, analysis of ROS levels in CCl_4_-induced acute liver injury using electrochemical microscopy not only implies inflammation-associated ROS but also correlates with cell death-mediated ROS in the intracellular space. These results highlight the drawbacks of conventional methods for indirect ROS/RNS measurement (e.g., fluorescent dyes), which are mostly focused on intracellular ROS production, while electrochemical microscopy allows the detection of both intracellular and extracellular total ROS/RNS levels.

### Oxidative stress in hepatectomy

**Fig. 6:**
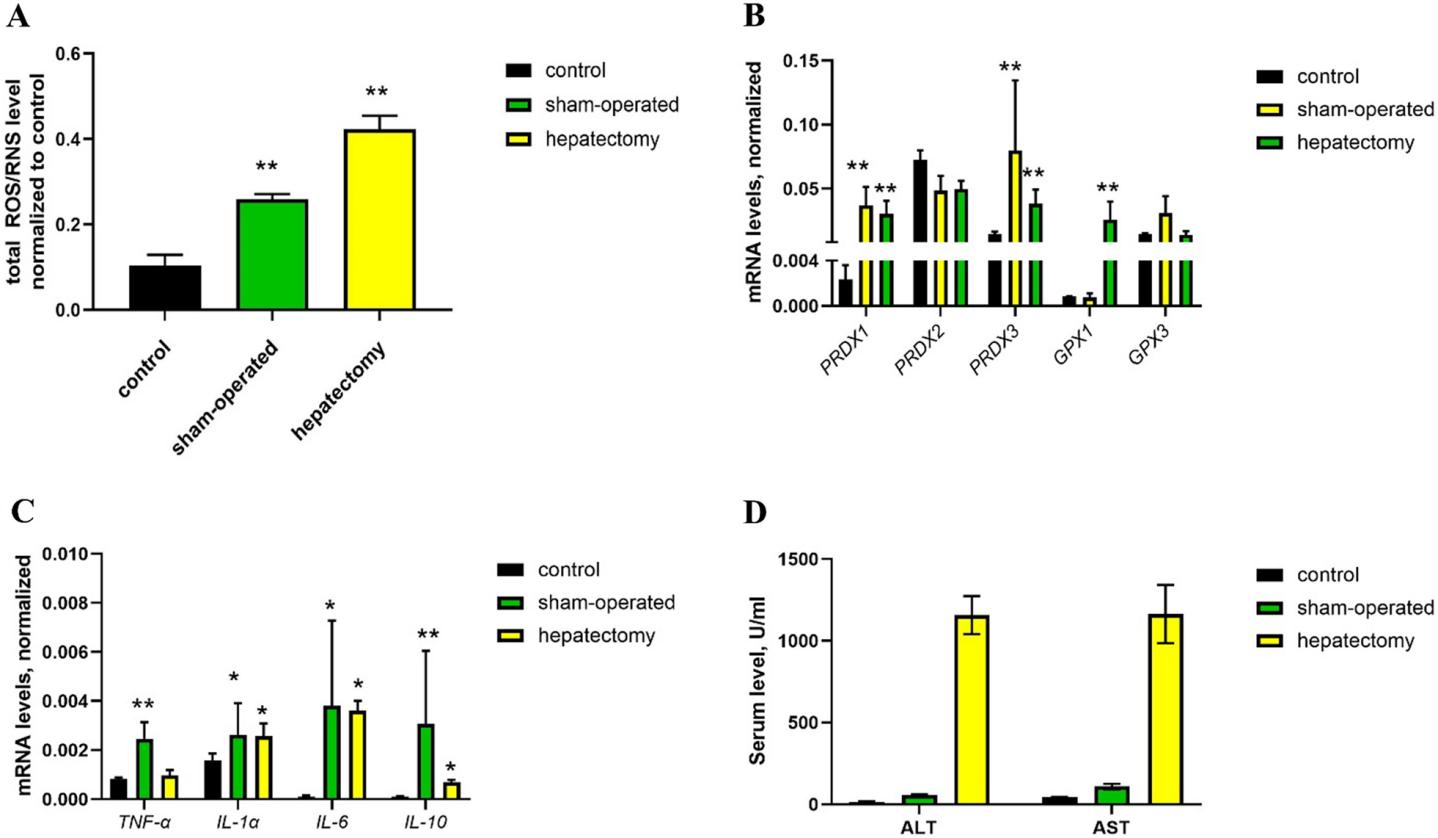
Analysis of the oxidative stress in mice after 30% partial hepatectomy. Measurement of total ROS/RNS levels using electrochemical microscopy. **A** Analysis of the expression of antioxidant defense enzymes and **B** proinflammatory cytokines **C** using RT-qPCR and serum ALT/AST levels. **D** Statistical analysis: **A**, **C** ** p-value < 0.001, * p-value < 0.005. **B**, **D** results show the mean ± SEM

Hepatectomy is a widely used model to study liver regeneration processes [35]. Partial hepatectomy leads to intense cell proliferation, inflammation and ROS/RNS bursts. The classic model relies on 70% hepatectomy, which leads to significant changes in liver morphology and protein expression patterns and great variability between the animals (especially for short-term experiments). We modified this model and performed a 30% hepatectomy to reduce these effects after surgery. As a proper control, we used sham-operated mice that passed the same procedures except removal of a liver lobe, including sanitation, abdomen cut, liver lobe rotation, saline instillation and wound closure by sutures. Using electrochemical microscopy, we found that even surgery procedures by themselves (without liver lobe removal) led to an increase in total ROS/RNS levels (Fig. 6A), activation of *PRDX/GPX* components of the antioxidant defense system (Fig. 4B) and proinflammatory cytokines (*TNF-α. IL-1α, IL-6, IL-10*, Fig. 4C). Although the serum ALT/AST level in mice after partial hepatectomy was significantly higher (at least 50X in comparison to control) (Fig. 6D), the sham-operated mice still demonstrated a small 2–3 fold increase, which confirms the development of inflammation 24 h after surgical manipulations.

**Fig. 7:**
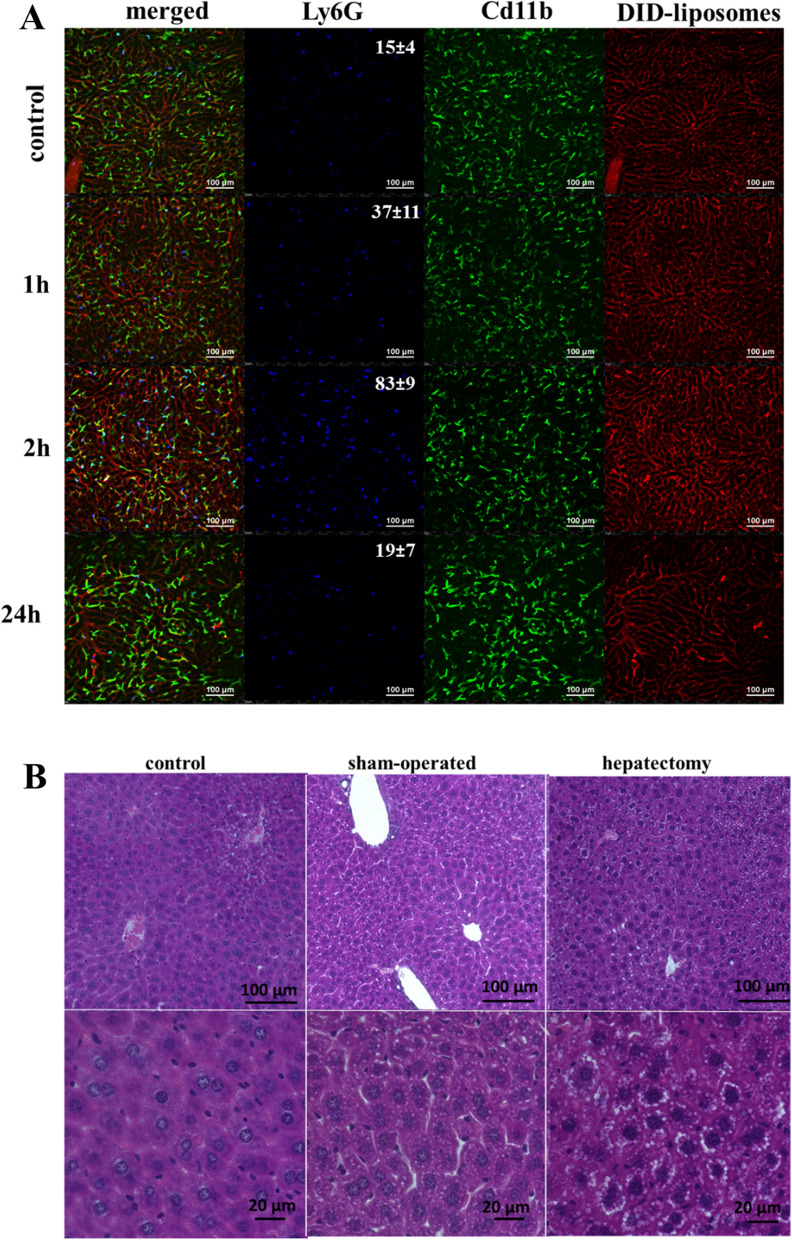
Pathological processes in the partial hepatectomy animal model. **A** Intravital imaging of the liver tissue at 1, 2 and 24 h after partial hepatectomy. The numbers of Ly6G + cells per field of view are shown as the mean ± SD. **B** Histological staining of liver tissue (hematoxylin–eosin) from control mice, sham-operated mice and mice after 30% partial hepatectomy

Using intravital confocal microscopy, we observed an increased fraction of neutrophils in the first hours after partial hepatectomy (Fig. 7A, 1 h-2 h). At 24 h after surgery, neutrophil counts returned to normal (Fig. 7A, 24 h), while hepatocytes were hypertrophied and had increased lipid and glycogen content (Fig. 7B). These processes precede the proliferation of the hepatocytes [36] and are highly associated with increased ROS/RNS. Therefore, the increase in ROS/RNS 24 h after partial hepatectomy is associated not only with inflammation after surgical manipulations but also with early-stage regeneration processes.

### Oxidative stress in hepatocellular carcinoma

**Fig. 8:**
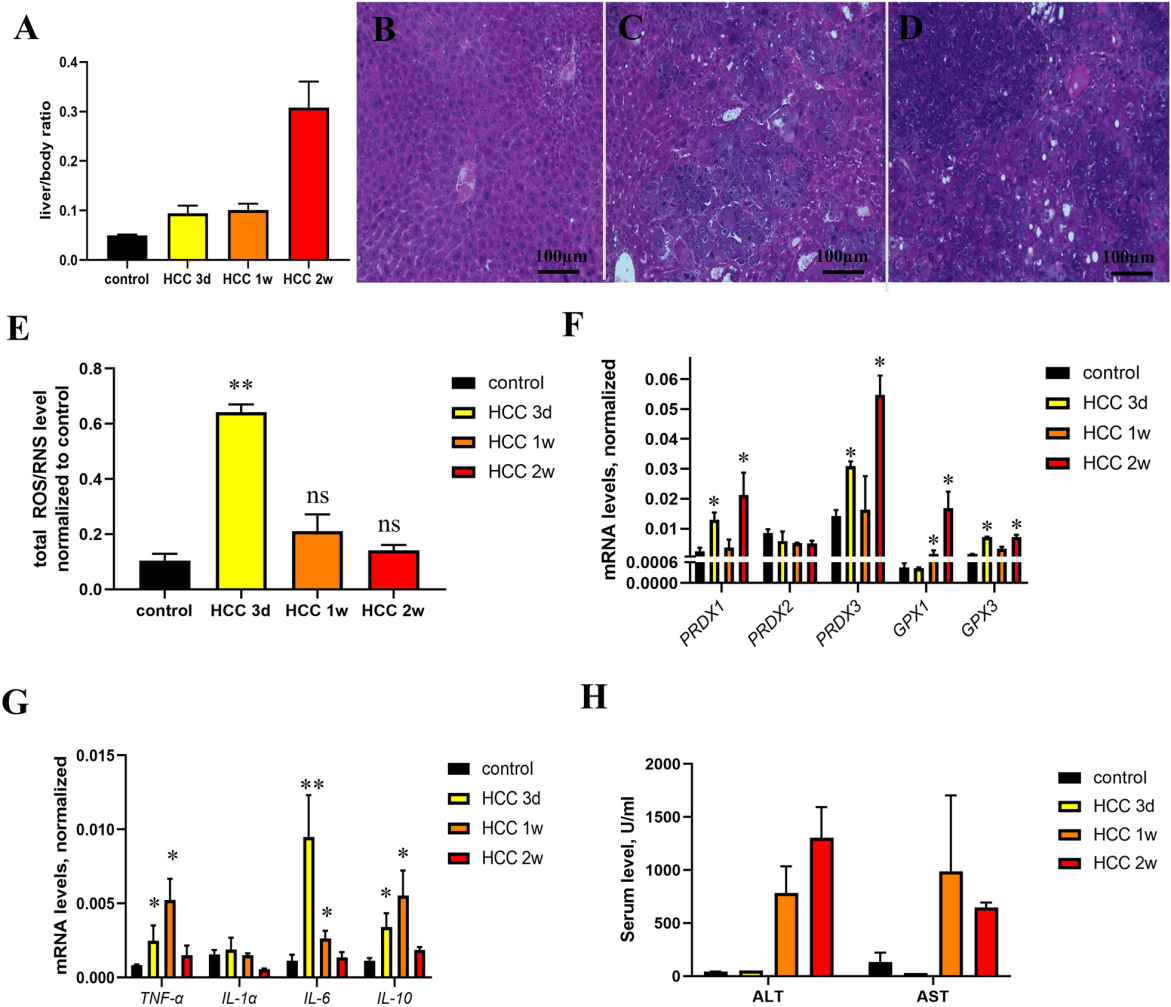
Analysis of oxidative stress in mice with hepatocellular carcinoma. Tumor growth of mice after hydrodynamic injection was analyzed by liver-body ratio (**A**) and histological hematoxylin–eosin staining at 3 days (**B**), 1 week (**C**) and 2 weeks (**D**) after injection of oncogenic plasmids. Measurement of total ROS levels using electrochemical microscopy (**E**), analysis of the expression of antioxidant defense enzymes (**F**) and proinflammatory cytokines (**G**) using RT-qPCR and serum ALT/AST levels (**H**). **p-value < 0.001

Cancer cells have increased ROS/RNS levels due to enhanced cell and mitochondrial metabolism [37]. Elevated ROS/RNS levels contribute to the development and growth of tumors. Here, we studied total ROS/RNS production in the liver 3, 7 and 14 days after the hydrodynamic injection of oncogenic plasmids. The liver-to-body ratio (Fig. 8A) and histological analysis showed a fast tumor growth rate for the 2-week model (Fig. 8B–D). Analysis of total ROS/RNS in tumors can be a challenging task due to the heterogenicity of the tissue. To evaluate this issue, we analyzed ROS/RNS at different depths and sites of the liver, but no significant difference was observed (Additional file 1: Figure S3). According to electrochemical microscopy data, maximal ROS levels were observed on day 3 after injection of oncogenic plasmids (Fig. 8E), while ALT/AST levels and changes in RT-qPCR data were maximal 2 weeks after injection (Fig. 8F-H). The expression of peroxiredoxins, glutathione reductase and cytokines increased from 3 to 14 days after tumor initiation (Fig. 6F, G). This difference can be a result of liver damage after hydrodynamic injection and poor ROS/RNS upregulation at the initial stages of tumor development [38]. It should also be noted that the heterogenicity of tumors leads to great variability between animals that decreases the significance of the results at later time points. Similar results were obtained using DCFDA analysis of ROS in this model (Additional file 1: Figure S9C).

## Discussion

Liver function, including the elimination of drugs and poisons, leads to significant changes in ROS/RNS levels within the organ [39]. Most liver diseases are associated with excessive production of ROS and chronic inflammation: nonalcoholic fatty liver disease, hepatitis, cirrhosis and, finally, hepatocellular carcinoma, from which a total of ~ 2 million people die per year [40]. Accurate and reliable quantification of ROS/RNS can simplify the assessment of drug toxicity in various organs and tissues, assessment of liver status before resection and analysis of liver regeneration before and after transplantation, assessment of embryo quality for in vitro fertilization, and much more [41, 42]. Safe and effective intravital measurements of total ROS/RNS levels in the liver can be a useful tool in preclinical studies and even in the clinic to improve the treatment of liver diseases. However, most modern methods are based on compounds sensitive to ROS, which can be oxidized during circulation and have a different background in the body due to different levels of ROS/RNS. For example, the intestine has high ROS/RNS levels and accumulates dyes due to their PK/PD properties, which makes it difficult to detect liver damage using ROS-induced fluorophores and whole-body fluorescence imaging [43]. In addition, the half-life of most sensors is limited, spectra can affect endogenous background fluorescence, and in vivo measurements depend on environmental and instrumental factors [44]. The interference of fluorescent dyes with different compounds and multiple troubleshooting were intensively studied to improve conventional methods [45]. Alternative techniques, such as intravital confocal microscopy, including the FLIM variant, require advanced surgical skills to expose as much of the organ as possible on a microscope slide [46]. The surface of the exposed part should be flat and away from the chest to avoid interference with chest movements (breathing, heartbeat) when receiving a fluorescent signal. At the same time, extensive surgical intervention leads to a strong influence of external factors (temperature, humidity, etc.) on the state of organs and is not applicable for long-term measurements in vivo. The duration of the measurement is critical for intravital ROS measurements. The long duration of the experiment can be associated with changes in cellular behavior [47, 48] and activation of the immune system (*i.e.* the penetration of neutrophils and macrophages as a source of ROS) and even the death of animals.

Electrochemical microscopy has previously been used to detect ROS/RNS. Wang et al. determined ROS and RNS (all four main species involved in oxidative stress) in solutions and murine macrophages using platinized nanoelectrodes. In our latest study, we exposed prostate cancer cells (22rv1 and PC-3) to doxorubicin (an anti-cancer drug and a well-known ROS inducer) and analyzed total ROS/RNS production in living cells using electrochemical microscopy. We also confirmed the data in mice with subcutaneous tumors and found increased levels of ROS/RNS in the tumor after intravenous administration of liposomal doxorubicin [21]. In this study, we developed a rapid minimally invasive technique to measure total ROS/RNS level in abdominal organs, particularly the liver. We explored the possibility of using electrochemical microscopy to quantify total ROS/RNS in normal and damaged livers in various animal models. First, we have demonstrated that we can obtain reproducible results for ROS/RNS levels at different sites or depths of the normal liver in vivo with no more than 5% variation in results. Since surgical manipulation can affect redox homeostasis, we have demonstrated that the signal does not change during at least 40 min of analysis. However, any manipulation led to an increase in total ROS/RNS levels, so we want to emphasize that any animal model used in ROS/RNS studies requiring surgical access must have proper control due to inflammatory responses.

To analyze ROS/RNS production in the damaged liver, we studied three models of liver pathologies associated with ROS/RNS imbalance in mice: CCl_4_-induced injury, partial hepatectomy, and hepatocellular carcinoma. These three models represent different processes that cause oxidative stress in the liver: massive cell death, inflammation, and cell hyperplasia, followed by proliferation, apoptosis, and transformation of cancer cells. In fact, we have demonstrated that all three models induce the expression of enzymes of the antioxidant defense systems and cytokines and increase ALT/AST levels. We also confirmed ROS production using a conventional fluorescent method – DCFDA assay. Using confocal microscopy and histological staining, we confirmed that the main source of ROS/RNS in the model of liver damage caused by CCl_4_ is not only intense neutrophil infiltration but also massive cell death. Necrotic processes are associated with intense ROS/RNS production (*i.e.*, extracellular [49, 50]), but dead cells cannot represent adequate redox status and can be analyzed using most conventional indirect measurements. In this experiment, we demonstrated the advantage of electrochemical microscopy over other methods in detecting total ROS/RNS levels in damaged livers after exposure to toxic agents. In addition to cell death, ROS/RNS can be formed during intensive cell proliferation [51]. We studied the hepatectomy model, as liver regeneration is accompanied by both cell proliferation and inflammation. We have shown that we can detect increased ROS/RNS levels associated with both processes. The detection of ROS/RNS in tumors is widely used in preclinical studies to predict the effectiveness of anticancer drugs [52]. However, the heterogeneity of tumor tissue and various antagonistic metabolic processes (intense proliferation and cell death) complicate the analysis of the real redox status. Using electrochemical microscopy, we were able to analyze total ROS/RNS level at different depths of liver tissue in the HCC model after hydrodynamic injection of oncogenic plasmids at different stages of tumor growth. The opportunity to quantify ROS at different depths could also be implemented for other fields of translational medicine in different heterogeneous processes (e.g., demyelination). In our model, we observed the highest ROS/RNS levels at day 3, which can be a result of liver injury, while later ROS/RNS levels decreased. Additionally, the heterogenicity of tumors led to great variability in animals and decreased the significance of the results between time points. Similar results were also obtained using a fluorescent DCFDA assay. These data can be a result of implicit tumor formation, as in the case of xenograft tumors, we are able to detect ROS/RNS after exposure to doxorubicin [21]. Also, another HCC model can differ in its characteristics (growth rate, intratumoral pressure, necrosis, hypoxia) that will reflect in greater heterogeneity of total ROS/RNS level.

Despite all the advantages of electrochemical microscopy, our setup has several limitations. First, foreign matter adhering to the sensor surface can interfere with or even block the signal. Second, this sensor can detect only total ROS/RNS without the opportunity to distinguish each compound. Further building the setup can solve this problem. In our opinion, electrochemical microscopy is a fast, minimally invasive method that is ideal for in vivo quantification of ROS/RNS in the liver. Compared to intravital confocal imaging and FLIM, electrochemical microscopy has several advantages: (1) direct measurement of ROS/RNS without any exogenous indicators; (2) the available depth for measurements exceeds 3000 µm and can be further increased using electrode technology that overcomes ~ 100 µm available for optical methods; (3) minimal invasiveness (simple and limited surgery), and (4) fast measurements (only 5–10 min for the whole experiment). Thus, electrochemical microscopy is an ideal solution for measuring total ROS/RNS levels in vivo and can become a promising tool for preclinical and clinical research.

## Supplementary Information

**Additional file 1. **Additional tables and figures.

## Data Availability

The datasets used and/or analysed during the current study are available from the corresponding author on reasonable request.
