# Supplementary material for: Intravital electrochemical nanosensor as a tool for the measurement of reactive oxygen/nitrogen species in liver diseases
Source: J Nanobiotechnology. 2022 Nov 24;20:497. doi: 10.1186/s12951-022-01688-z (PMC9694853; doi:10.1186/s12951-022-01688-z)
Supplement: Supplementary file 1 — Additional file 1. Additional tables and figures. [file 12951_2022_1688_MOESM1_ESM.docx]

**Intravital electrochemical nanosensor as a tool for the measurement of reactive oxygen/nitrogen species in liver diseases**

Tatiana Abakumova^1,6^**^ǂ^***, Alexander Vaneev**^ǂ^***^2,7^, Victor Naumenko^3^, Arina Shokhina^4^, Vsevolod Belousov^4^, Arsen Mikaelyan^5^, Kamilla Balysheva  ^2^*,* Peter Gorelkin^2^, Alexander Erofeev^2,7^, Timofei Zatsepin^7^

**Additional information**

**Serum analysis (ALT/AST level)**

Serum alanine-transferase (ALT) and aspartate-transferase (AST) were analyzed by HTI Biochem Analytte (High Technology, USA). Serum from nontreated mice was used as a control.

**RT-qPCR**

Analysis of mRNA levels for antioxidant defense, inflammation and cell proliferation markers was performed using RT-qPCR. Briefly, RNA was isolated from liver tissue using TRIzol™ (Invitrogen, #155960) according to the manufacturer’s instructions. cDNA was synthesized using a Maxima First Strand cDNA Synthesis Kit (ThermoFisher, K1641). Levels of cDNA were assessed by qPCR using PowerUp™ SYBR™ Green Master Mix (Applied Biosystems™ A25742) in the CFX96 Touch Real-Time PCR Detection System (Bio-Rad). mRNA levels were normalized to the housekeeping gene (mGAPDH) and the average value of the control group. Specific primers are listed in Supplementary Table S2.

**Intravital confocal microscopy**

Liver preparation for IVM was performed as previously described [50]. In brief, mice were anesthetized by intraperitoneal injection of zoletil (50 mg/kg) and xylazine (5 mg/kg). To expose the liver, a midline incision was made followed by a lateral incision along the costal margin to the midaxillary line. The mouse was placed in a right lateral position, and the ligaments attaching the liver to the diaphragm and the stomach were cut, allowing the liver to be externalized onto a glass coverslip on the inverted microscope stage (Figure S4). Fluorescently labeled antibodies (0.04 mg/kg Ly6G BV421 (clone 1A8) and 0.07 mg/kg CD11b PE (clone M1/70), Biolegend) were intravenously injected to stain neutrophils and Kupffer cells. Sinusoids were counterstained by DiD-labeled liposomes. IVM was performed on a Nikon A1R confocal microscope using a Plan Apo 20×/0.75 DIC N objective (numerical aperture 0.75; Nikon Japan) and Apo LWD 40×/1.15 S water immersion objective (numerical aperture 1.15; Nikon Japan). Images were scanned sequentially using 405-, 488-, 561- and 647-nm diode lasers in combination with a DM405/488/561/633 nm dichroic beam splitter. Imaging analysis and quantification were performed using NIS Elements AR software. For each liver sample, the counts of Ly6G+ cells and CD11b+ cells were analyzed in 8–10 random fields of view (640 ×640 μm).

**Histological staining**

Liver samples were fixed in 10% neutral formalin solution (Sigma, HT501128) and embedded in paraffin using standard procedures. Five-micrometer-thick sections were subjected to hematoxylin-eosin (H&E) staining. The H&E sections were analyzed using a Keyence BZ-9000 microscope with 20x and 60x magnification.

**DCFDA assay in liver homogenates.**

Analysis of total ROS in liver homogenates was performed using 2’,7’-dichlorofluorescein reagent (H2DCFDA, Life Technologies). First, we added ice-cold Tris-HCl buffer (40 mM, pH 7.4) to snap-frozen liver samples in homogenization tubes (1 ml per 100 mg of liver tissue). After homogenization, the samples were centrifuged (4000 rpm, 5 min), and the supernatant was dissolved 10 times in Tris-HCl buffer (40 mM, pH 7.4) in amber 1.5 ml tubes. After that, a freshly prepared solution of H2DCFDA (10 mM in DMSO) was added to the liver samples (5 µl per 1 ml of dissolved liver homogenate) and analyzed in a microplate reader. Fluorescence intensity was analyzed using Varioskan Lux at 485/520 excitation/emission spectrum immediately and for a subsequent 30 min at 37°C. Liver samples from nontreated animals were used as controls.

**Table S1. Recent advances in sensing markers of oxidative stress**

| Method | Organ | Analyte | Advantages / Disadvantages | Ref. |
| --- | --- | --- | --- | --- |
| Electron paramagnetic resonance (EPR) | Liver (ex vivo) | ROS | + detection of unstable reactive species   - lack of selectivity for the detection of one particular type of radical - only *ex vivo*/ *in vitro* experiments - special expensive equipment | [5] |
| Fluorescent method | Liver | H_2_O_2_, GSH, HOCl | + simultaneous detection of ROS and GSH  + wide dynamic range of sensitivity  - irreversibility of probes  - short time window of fluorescence-lifetime imaging microscopy | [42] |
| Combined fluorescent and chemilumi-nescent method | Liver (*in vivo*) | H_2_O_2_, ONOO^-^ | + simultaneous and differential dual-analyte sensing using two optical channels  + excellent photostability  + high specificity   - complex fluorescent probe preparation | [51] |
| Electroche-mical method | In vitro model (Lab-on-chip) | ROS | + good in vitro model for research of fibrosis development  - inability to reproduce communication with other organs  - high complexity of experiment and high cost | [52] |
| Electrochemi-cal method | Liver (*ex vivo*) | NO | + long-term measurements during several days  + highly temporary resolution  + wireless transmission  - the need for surgery to mount the electrode | [27] |
| Electrochemi-cal method | 4T1 и MC4L2 tumor  *(in vivo)* | H_2_O_2_ | + detection of the vital or necrosis state of the tumors  - relatively invasive method | [28] |
| Electrochemi-cal method | Rat parietal cortex  (*in vivo*) | NO | + Long-term measurements  + Measurement of absolute concentrations  + High selectivity to NO   - Basal NO concentration was not detectable | [26] |
| Electrochemi-cal method | 4T1 Tumor (*in vivo*) | ROS | + Minimally invasive technique  + Highly sensitive method  + Highly temporary resolution  + Possibility of ROS detection inside deep tissue layers  + Long-term measurements   - Contamination of the surface of electrode with biomolecules (proteins, oxidation products, etc.) | [21] |
|  |  |  |  |  |

**Table S2. Specific primers for qPCR used in the study**

|  |  | Forward primer | Reverse primer |
| --- | --- | --- | --- |
| Prdx1 |  | AATGCAAAAATTGGGTATCCTGC | CGTGGGACACACAAAAGTAAAGT |
| Prdx2 |  | GATGGTGCCTTCAAGGAAATCA | CCGTGGGGCAAACAAAAGTG |
| Prdx3 |  | GGTTGCTCGTCATGCAAGTG | CCACAGTATGTCTGTCAAACAGG |
| Gpx1 |  | CCACCGTGTATGCCTTCTCC | AGAGAGACGCGACATTCTCAAT |
| Gpx3 |  | CCTTTTAAGCAGTATGCAGGCA | CAAGCCAAATGGCCCAAGTT |
| TNFa |  | CCACCACGCTCTTCTGTCTA | TCGAATTTTGAGAAGATGATCTGAG |
| IL1a |  | AAGTCTCCAGGGCAGAGAGG | ACTGTAGTCTTCGTTTTCACTGT |
| IL6 |  | TCCAGTTGCCTTCTTGGGAC | GCCATTGCACAACTCTTTTCTCA |
| IL10 |  | GGTTGCCAAGCCTTATCGGA | GAGAAATCGATGACAGCGCC |
| TGF-β |  | AAATCAACGGGATCAGCCCC | CGCACACAGCAGTTCTTCTC |
| IFNγ |  | AAGACAATCAGGCCATCAGCA | TGTGGGTTGTTGACCTCAAACT |
| IL18 |  | GGCTGCCATGTCAGAAGACT | ACAGTGAAGTCGGCCAAAGT |


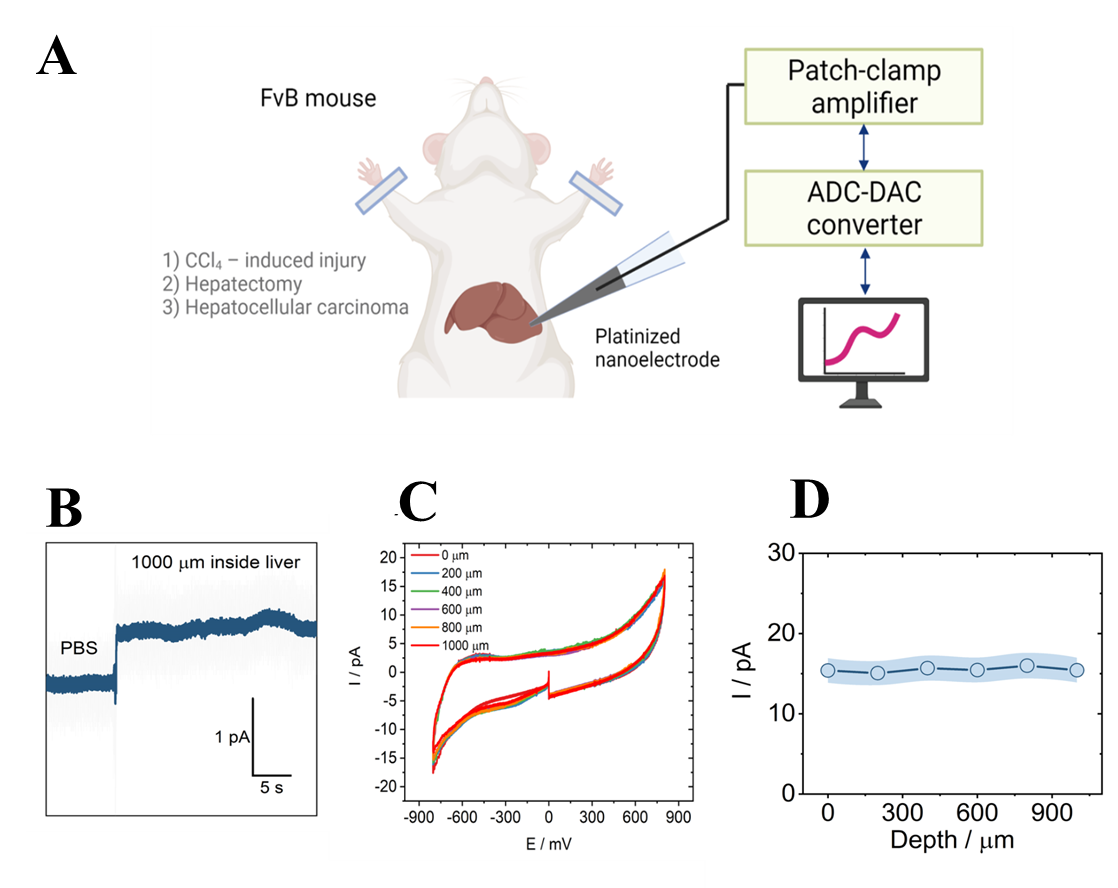


Figure S1. Electrochemical microscopy of the liver tissue. The setup for ROS/RNS measurements in the liver


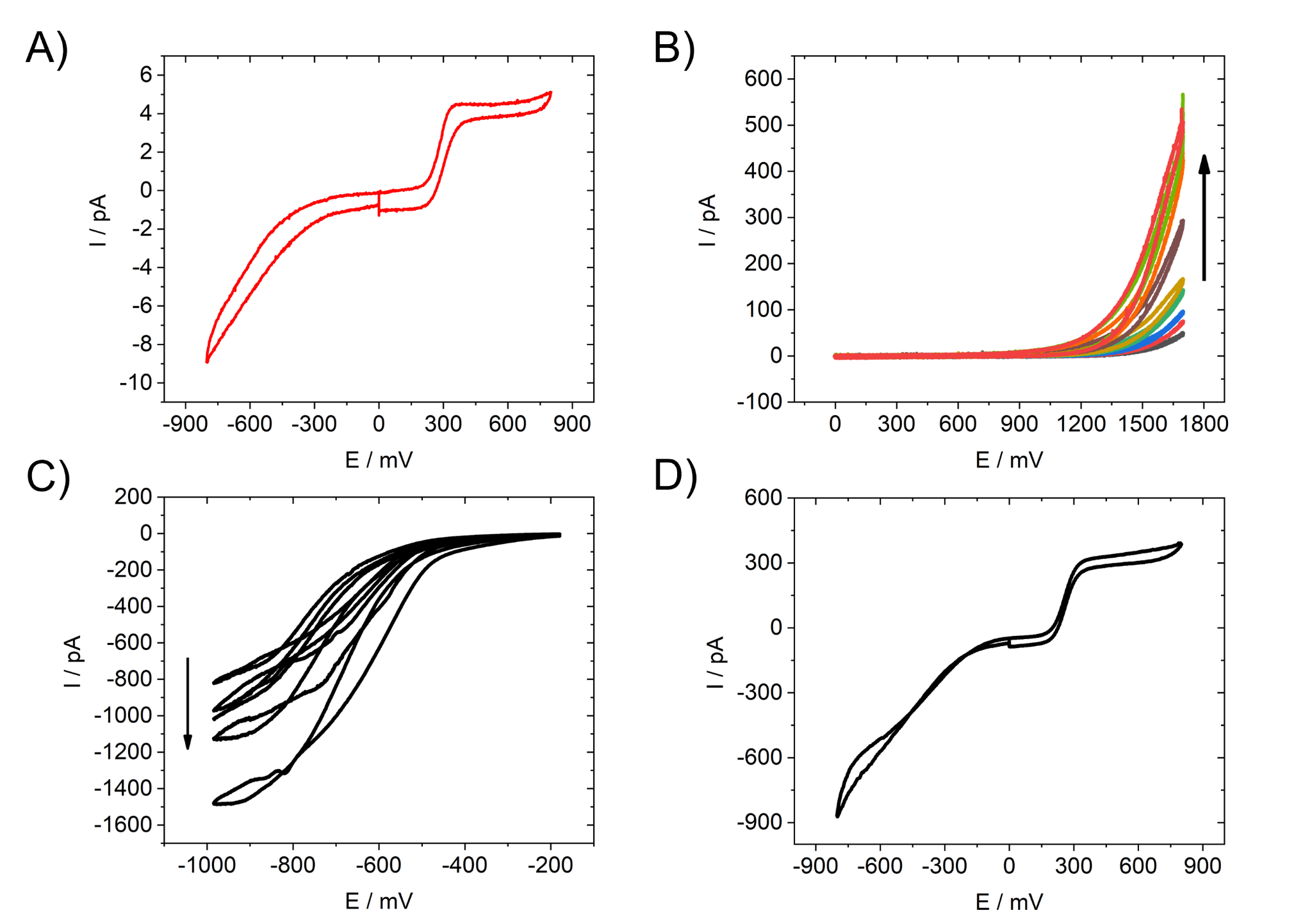


Figure S2. Fabrication and evaluation of Pt nanoelectrodes. (A) CV of the carbon nanoelectrode in 1 mM ferrocene methanol in PBS. Sweep rate 400 mV s-1 (B) Current-voltage characteristic was recorded during the process of etching the carbon nanoelectrode in a 0.1 M NaOH, 10 mM KCl solution to create a cavity on the nanoelectrode surface. (C) Current-voltage characteristic was recorded during the platinum deposition process carried out by sweeping the potential from 0 to −800 mV vs Ag/AgCl in a solution containing 2 mM H2PtCl6. (D) CV of platinum nanoelectrode in 1 mM ferrocene methanol in PBS. Sweep rate 400 mV s-1

^
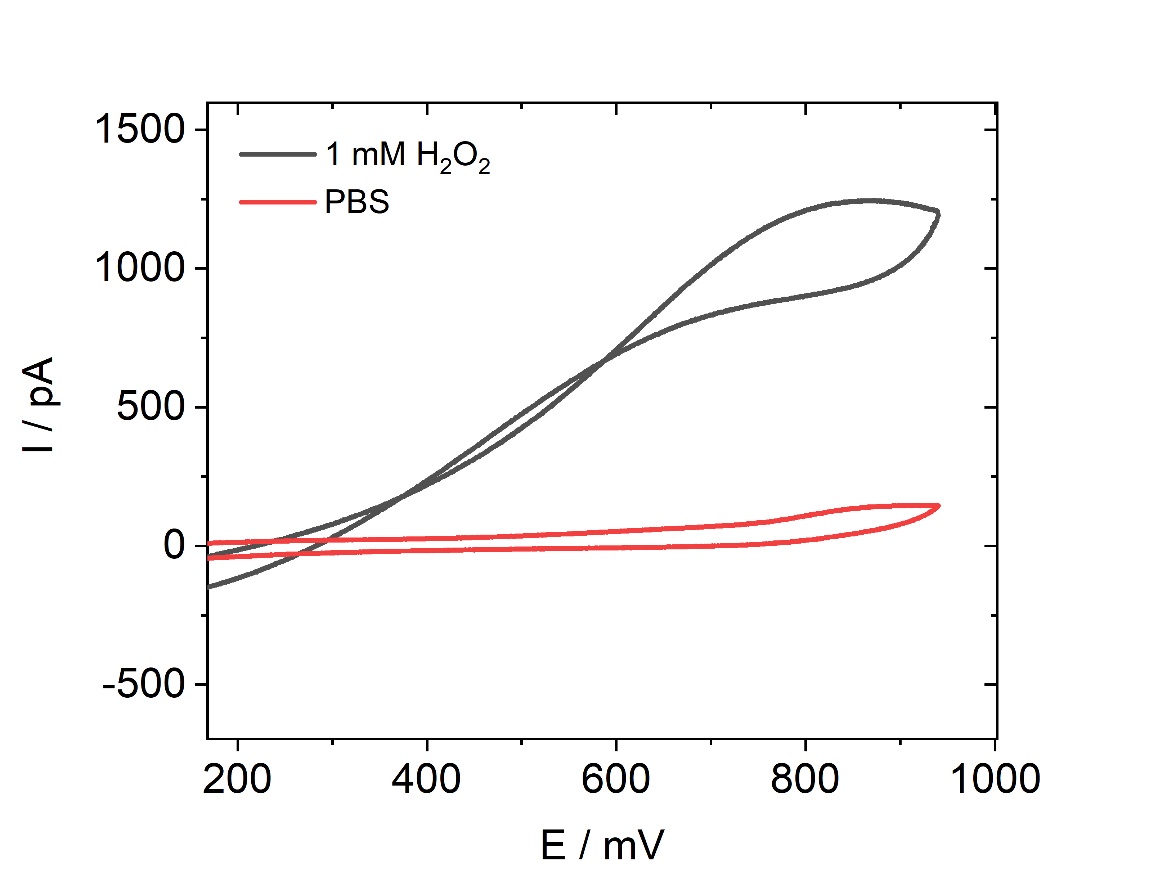
^

Figure S3. CV of the Pt nanoelectrode in 1 mM H_2_O_2_ in PBS. Sweep rate 400 mV s^-1^


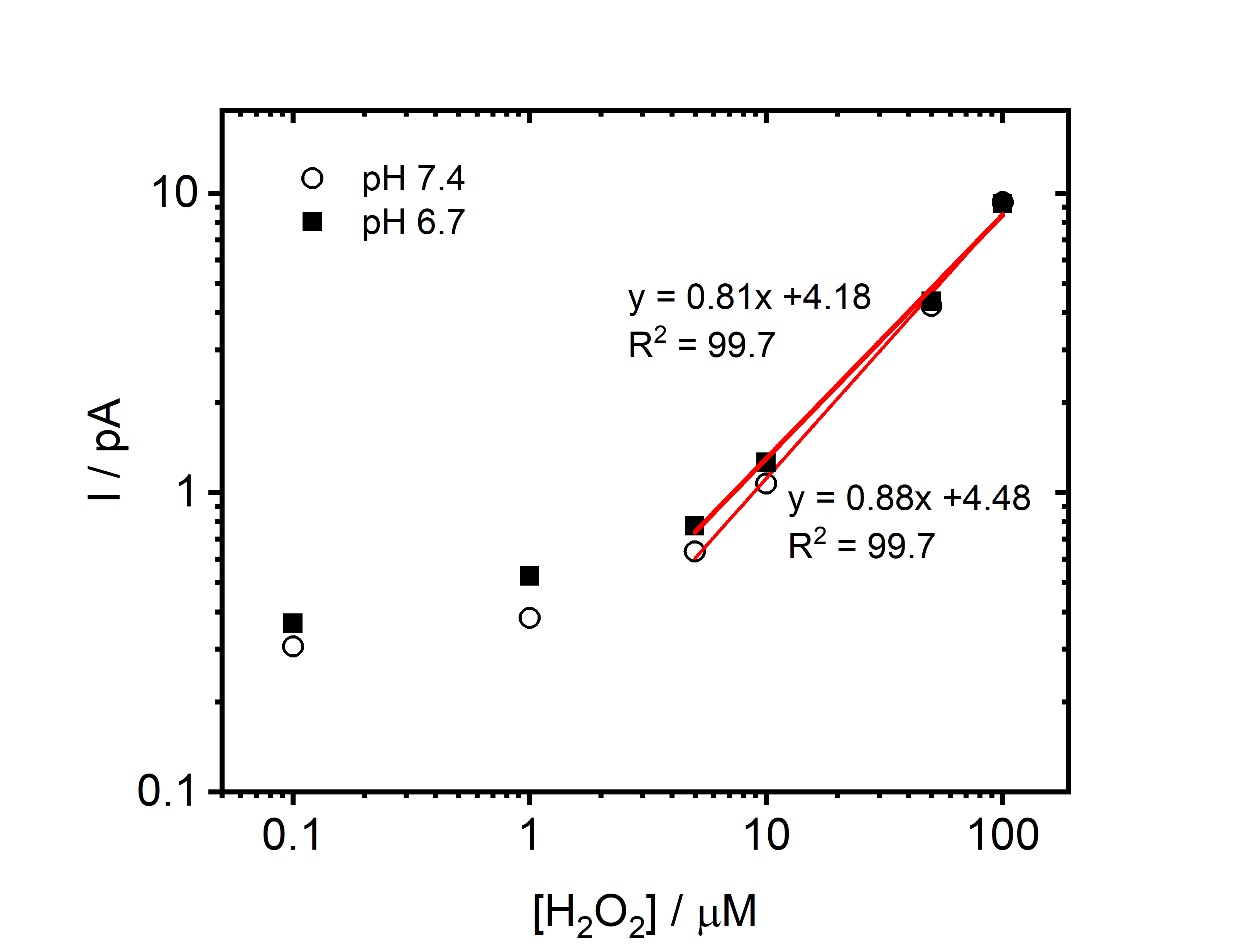


Figure S4. Calibration curve (current vs H_2_O_2_ concentration at +800 mV (vs. Ag/AgCl)) in pH 6.7 and 7.4


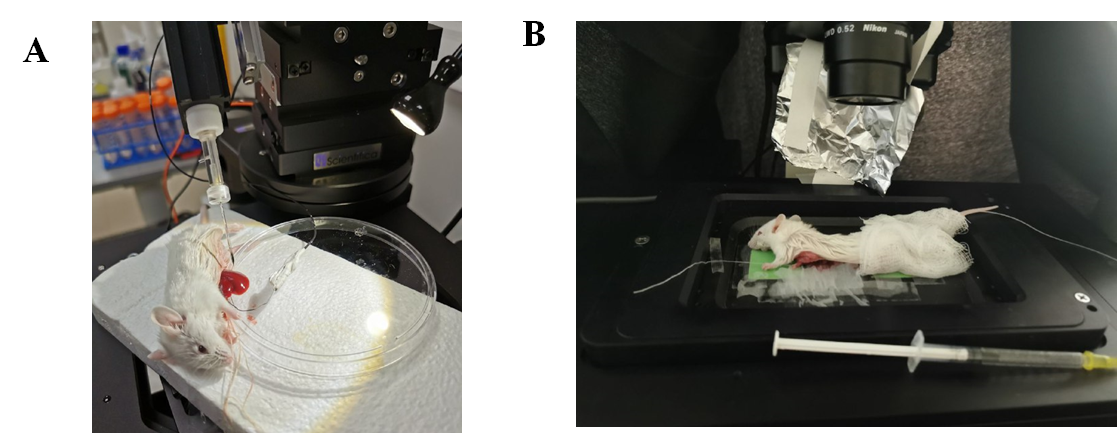
Figure S5. Process of measurements *in vivo* using electrochemical (A) or confocal imaging (B).


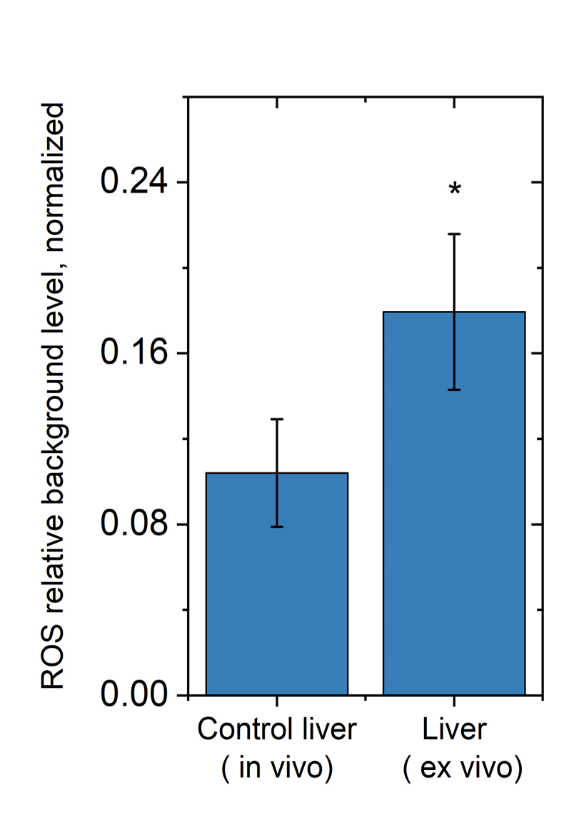


Figure S6. Analysis of total ROS/RNS levels by electrochemical microscopy in normal livers in living animals (in vivo) and in isolated organs (ex vivo). * p-value <0.05


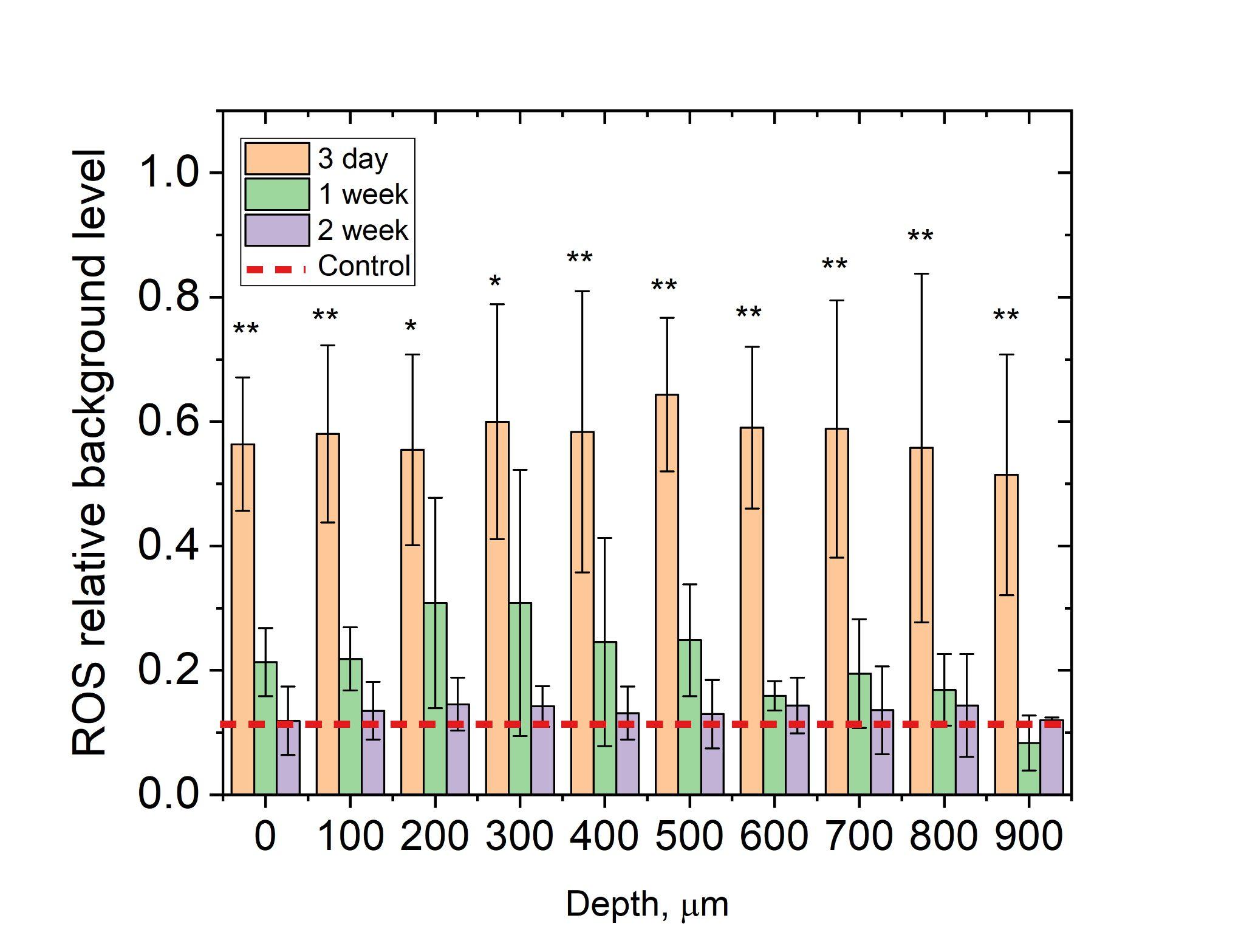


Figure S7. Analysis of total ROS/RNS levels at different depths of tumor tissue at 3 days, 1 week and 2 weeks after hydrodynamic injection. **p-value<0.001, **p-value<0.005


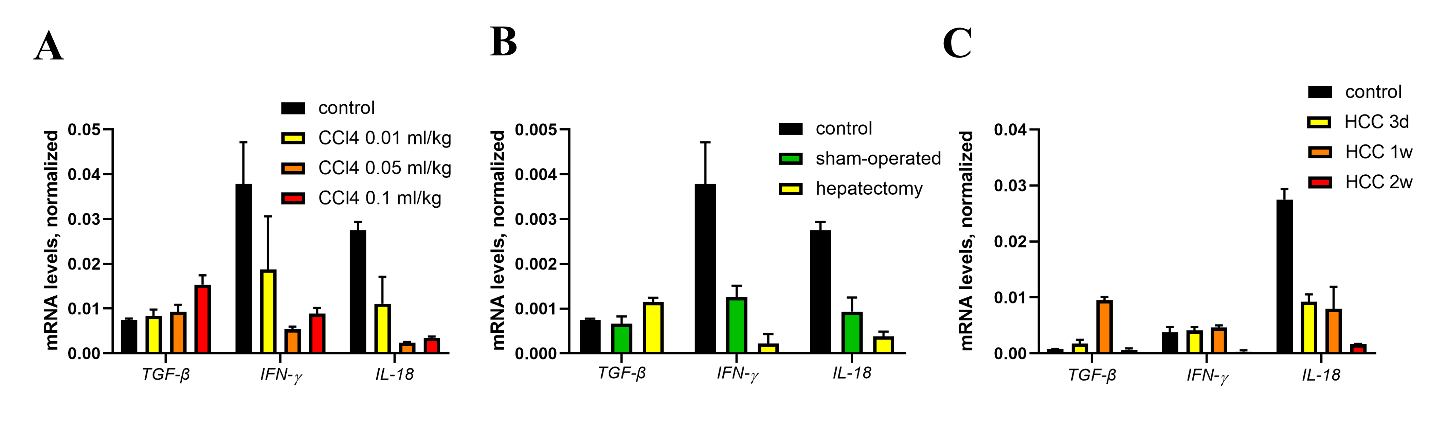
 Figure S8. Analysis of the mRNA levels of cytokines in different liver disease models: (A) ССl_4_-induced liver injury, (B) partial hepatectomy and (С) hydrodynamic model of hepatocellular carcinoma. Data are presented as the mean±SEM.


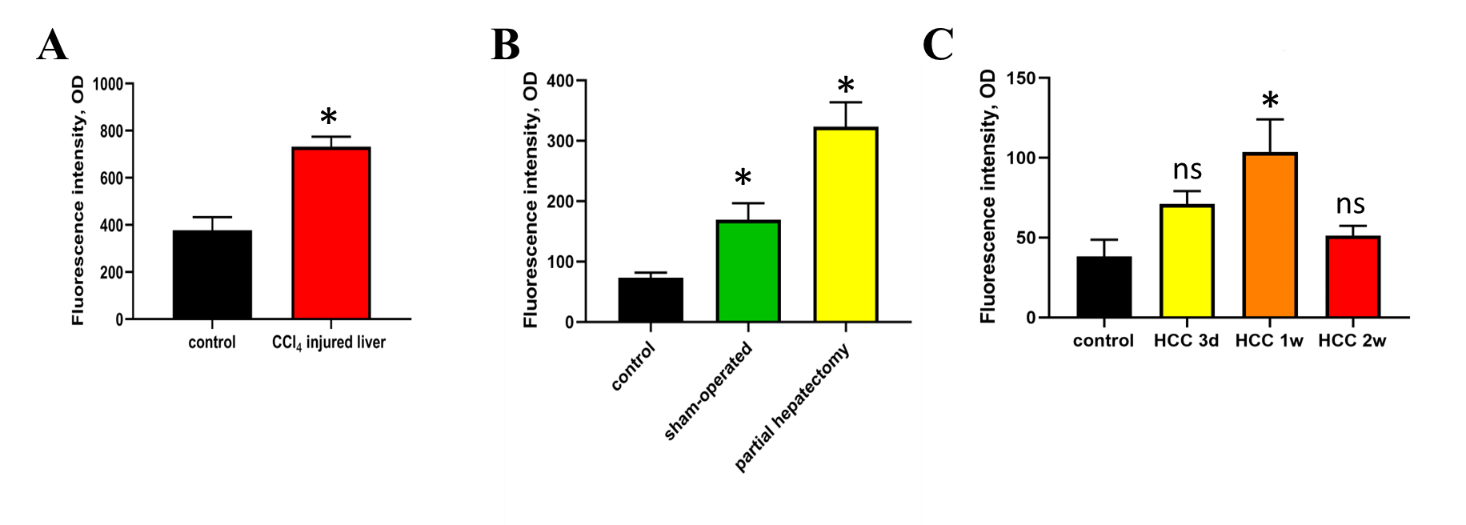


Figure S9. Analysis of total ROS using dichlorofluorescein (DCFDA assay) in liver tissue homogenates in different liver disease models: (A) ССl_4_-induced liver injury, (B) partial hepatectomy and (С) hydrodynamic model of hepatocellular carcinoma. Data are presented as the mean±SEM. *-p-value<0.005, ns- nonsignificant.
